# Internal exposure to heat-induced food contaminants in omnivores, vegans and strict raw food eaters: biomarkers of exposure to 2- and 3-monochloropropanediol (urinary excretion) and glycidol (hemoglobin adduct *N*-2,3-dihydroxypropyl-Val)

**DOI:** 10.1007/s00204-024-03880-6

**Published:** 2024-10-01

**Authors:** Bernhard H. Monien, Jan Kuhlmann, Fabian Gauch, Cornelia Weikert, Klaus Abraham

**Affiliations:** 1 Department of Food Safety, German Federal Institute for Risk Assessment (BfR), Max-Dohrn-Strasse 8-10, 10589 Berlin, Germany; 2 SGS Germany GmbH, Weidenbaumsweg 137, 21035 Hamburg, Germany

**Keywords:** Human exposure, 2-MCPD, 3-MCPD, Glycidol, Hemoglobin adducts, Biomarker

## Abstract

Fatty acid esters of 2/3-monochloropropanediol (2/3-MCPD) and glycidol are formed mainly during heat processing (deodorization) of vegetable oils, and are hydrolyzed by lipases in the gastrointestinal tract leading to the absorption of 2/3-MCPD and glycidol. The International Agency for Research on Cancer (IARC) has classified 3-MCPD as possibly and glycidol as probably carcinogenic to humans. The aims of the current work were to clarify the exposure to 2/3-MCPD and glycidol associated with different dietary habits (omnivore, vegan, raw-food eating), and the exposure development between 2017 and 2021 in German study participants. The questions were addressed using the daily urinary excretion of 2/3-MCPD and the hemoglobin adduct *N*-(2,3-dihydroxypropyl)-Val (DHP-Val) formed from glycidol as biomarkers of exposure, which were determined in two dietary studies including 36 omnivores, 36 vegans and 16 strict raw food eaters (abstaining from any heated food for at least four months). The median urinary excretion of 2- and 3-MCPD in non-smoking omnivores and vegans was 0.87 and 1.35 µg/day (2-MCPD), respectively, and 0.79 and 1.03 µg/day (3-MCPD), respectively. The 2/3-MCPD concentrations in urine samples of raw food eaters were usually below the limit of detection. The median DHP-Val levels in non-smoking vegans and omnivores were 3.9 pmol/g Hb each, and 1.9 pmol/g Hb in raw food eaters. Between 2017 and 2021, the exposure to 3-MCPD and glycidol did not change, however, the median 2-MCPD excretion decreased (*p* = 0.02, omnivores and vegans combined). The correlation between daily excretions of 2/3-MCPD determined 4 years apart was weak, whereas a moderate correlation was observed for DHP-Val (*r*_S_ = 0.66) in this timeframe. In conclusion, the exposure to glycidol in omnivores and vegans was alike, whereas the 2/3-MCPD exposure was somewhat (albeit not significantly) higher in vegans. While 2/3-MCPD were hardly detectable in urine samples of raw food eaters, the median DHP-Val level (about 50% of those in omnivores) indicates a glycidol source independent of the dietary exposure.

**Supplementary Information:**

The online version contains supplementary material available at 10.1007/s00204-024-03880-6.

## Introduction

About 20 years ago, the fatty acid esters of different C3-compounds, i.e. glycidol, 2-monochloro-1,3-propanediol (2-MCPD) and 3-monochloro-1,2-propanediol (3-MCPD), were discovered as common food contaminants in refined oils and fats, in which they are formed during heat processing (deodorization) (Kuhlmann 2011; Weisshaar and Perz 2010; Zelinkova et al. 2006). Bioavailability studies in rats treated with 3-MCPD dipalmitate, glycidyl palmitate or glycidyl linoleate showed that the esters are hydrolyzed nearly quantitatively by lipases in the gastrointestinal tract to yield 3-MCPD (Abraham et al. 2013; Barocelli et al. 2011) as well as glycidol (Appel et al. 2013; Wakabayashi et al. 2012), leading to a more or less complete absorption.

The toxicity profiles of all three compounds are different. The most important neoplastic effect of 3-MCPD observed in 2-year bioassays was the increased incidence of tubular adenomas and carcinomas in both sexes of mice and rats (Cho et al. 2008; Sunahara et al. 1993). There is no evidence that 3-MCPD is genotoxic in vivo in any organ tested (Aasa et al. 2017b; El Ramy et al. 2007; Onami et al. 2014). The metabolite(s) promoting the toxic/carcinogenic effects or the modes of action are still not characterized (Eisenreich et al. 2023). The structural isomer 2-MCPD induced myopathy in striated muscles cells and nephrotoxicity in a 28-day study (Perrin et al. 1994). Glycidol induced tumors in multiple tissues, e.g., mesotheliomas in the tunica vaginalis and peritoneum and brain gliomas (in rats) and neoplasms of the Harderian gland, the forestomach and the mammary gland (in mice), in a 2-year bioassay (National Toxicology Program 1990). The reactivity with DNA and the mutagenicity in a variety of in vitro and in vivo tests led to the classification as a genotoxic carcinogen (Bakhiya et al. 2011).

Based on the data from animal studies, the International Agency for Research on Cancer (IARC) has classified the compounds as possibly (3-MCPD) and probably (glycidol) carcinogenic to humans (IARC 2013). The risk assessment of human exposure of 3-MCPD by the European Food Safety Authority (EFSA) is based on the consideration that 3-MCPD is not genotoxic in vivo. A tolerable daily intake (TDI) of 2 µg/kg body weight (bw) was calculated for the intake of 3-MCPD (or molar equivalents of the fatty acid esters) from the most sensitive endpoint, i.e. the increased incidence of kidney tubular hyperplasia in male rats (BMDL_10_ 0.2 mg/kg bw/day) (EFSA 2018). Median estimates of dietary exposure in different age groups (ranging from 0.2 to 0.7 μg/kg bw/day in *adults*, *elderly*, and *very elderly*) in Europe from 2016 were well below the TDI. However, the mean daily exposure was relatively high (0.5–1.5 μg/kg bw/day) for *infants (*< 12 months old), *toddlers* (≥ 12 months to < 36 months old), and *other children* (≥ 36 months to 10 years old) and exceeded the TDI for *infants receiving formula only* (2.4 μg/kg bw/day). The toxicity data were deemed to be insufficient for the risk assessment of 2-MPCD (EFSA 2016).

Due to the genotoxicity of glycidol, the margin of exposure (MoE) concept (EFSA 2005) was applied to assess the carcinogenic risk by comparing the daily human exposure with the dose that induces a significant increase of tumor incidence in a 2-year bioassay in animals. The MoE was calculated from the T25, i.e. the daily dose that increases the incidence of a specific tumor above background in the lifespan by 25% (10.2 mg/kg bw/day for the induction of peritoneal mesothelioma in male rats). The median values across the dietary surveys reporting the mean chronic exposure to glycidol in European countries were in the range of 0.1 to 0.5 μg/kg bw/day for *adolescents* and adult population groups, resulting in MoE values between 20,400 and 102,000. The median exposure for *infants*, *toddlers* and *other children* was in the range of 0.6 to 0.7 µg/kg bw/day resulting in MoEs between 14,800 and 17,000. In the group of *infants receiving formula only* the estimated mean daily intake was 1.9 µg/kg bw/day (MoE 5,500). Using the T25, the MoEs lower than 25,000 indicate a health concern (EFSA 2016; German Federal Institute for Risk Assessment 2009).

Measures have been taken to reduce the exposure of 2/3-MCPD, glycidol and their fatty acid esters. Food industry has worked on strategies to avoid the formation and to reduce the occurrence of the compounds (Oey et al. 2019; Yung et al. 2023). Regulation in particular has focused on infants and young children by implementation of maximum levels in various food stuffs. For example, EU maximum levels of 3-MCPD (free and bound) in infant formulae sold as powder or as liquid are 125 and 15 µg/kg, respectively. From January 2025 on, the values will be 80 and 12 µg/kg, respectively (European Commission 2024). EU maximum levels for glycidyl fatty acid esters (expressed as glycidol) in vegetable oils and fats, fish oils and oils from other marine organisms placed on the market for the final consumer are 1,000 µg/kg and in infant formulae sold as powder or as liquid 50 and 6.0 µg/kg, respectively (European Commission 2023).

Considering the joint efforts of mitigation by food industries and of legislation, it can be expected that the human dietary exposure to 2/3-MCPD and glycidol may have decreased and may further decrease in the future. This can be easily monitored using biomarkers of internal exposure to heat-induced food contaminants, e.g., hemoglobin (Hb) adducts or urinary metabolites. Hb adducts are medium-term biomarkers, reflecting the mean exposure over a few months, while metabolites in 24-h urine are short-term biomarkers. They represent the exposure on the day before and on the day of the urine collection. The exposure to glycidol can be monitored using the Hb adduct 2,3-dihydroxypropyl-Val (DHP-Val) (Abraham et al. 2019; Landin et al. 2000; Monien and Abraham 2023). A controlled exposure study was conducted by Abraham et al. (2019), in which adult participants (n = 11) consumed defined amounts of palm fat with a high content of glycidyl esters over 28 days. The additional individual doses between 2.7 and 5.2 μg/kg bw/day (mean 4.2 μg/kg bw/day) led to a weekly monitored increase of DHP-Val levels. The data allowed calculating a ratio between DHP-Val levels and glycidol dose, which in turn makes it possible to estimate the daily glycidol exposure of the last months from a single measurement of DHP-Val in Hb. The daily exposure to 2/3-MCPD as well as their fatty acid esters is monitored by the urinary excretion of 2/3-MCPD themselves (Abraham et al. 2021). A study with 12 adult participants exposed to a hazelnut oil with known high amounts of 2/3-MCPD fatty acid esters led to a mean urinary excretion of 14.4% 2-MCPD and 3.7% 3-MCPD. These values can be used as conversion factors to estimate the daily oral intake of 2/3-MCPD (free or in form of fatty acid esters) from the excretion of 2/3-MCPD in 24-h urine (Abraham et al. 2021).

In the current work, two main questions were addressed. First, we wanted to clarify the influence of different dietary habits (omnivore, vegan, raw-food eating) on the exposure to 2/3-MCPD and to glycidol. The urinary excretion of 2/3-MCPD and DHP-Val levels in Hb were analyzed in samples collected in the Risks and Benefits of a Vegan Diet (RBVD) study, which included 36 omnivores and 36 vegans (Weikert et al. 2020). Sixteen raw food eaters strictly avoiding consumption of food stuffs heated to higher temperatures than 42 °C were recruited in a separate study (Abraham et al. 2022). The latter study group was of particular interest to investigate a possible exposure apart from that through heat-treated food. Such an exposure has been observed for acrylamide and was attributed to an endogenous formation (Goempel et al. 2017; Monien et al. 2024). Second, the exposure development between 2017 and 2021 in German study participants was studied with both biomarkers, in order to test whether there is a relevant downtrend of exposure in this time frame resulting from mitigation and regulation. Further it was of interest to investigate the stability over time of the biomarker levels from the perspective of individual study participants.

## Materials and methods

### Study populations and sample collection

**Fig. 1:**
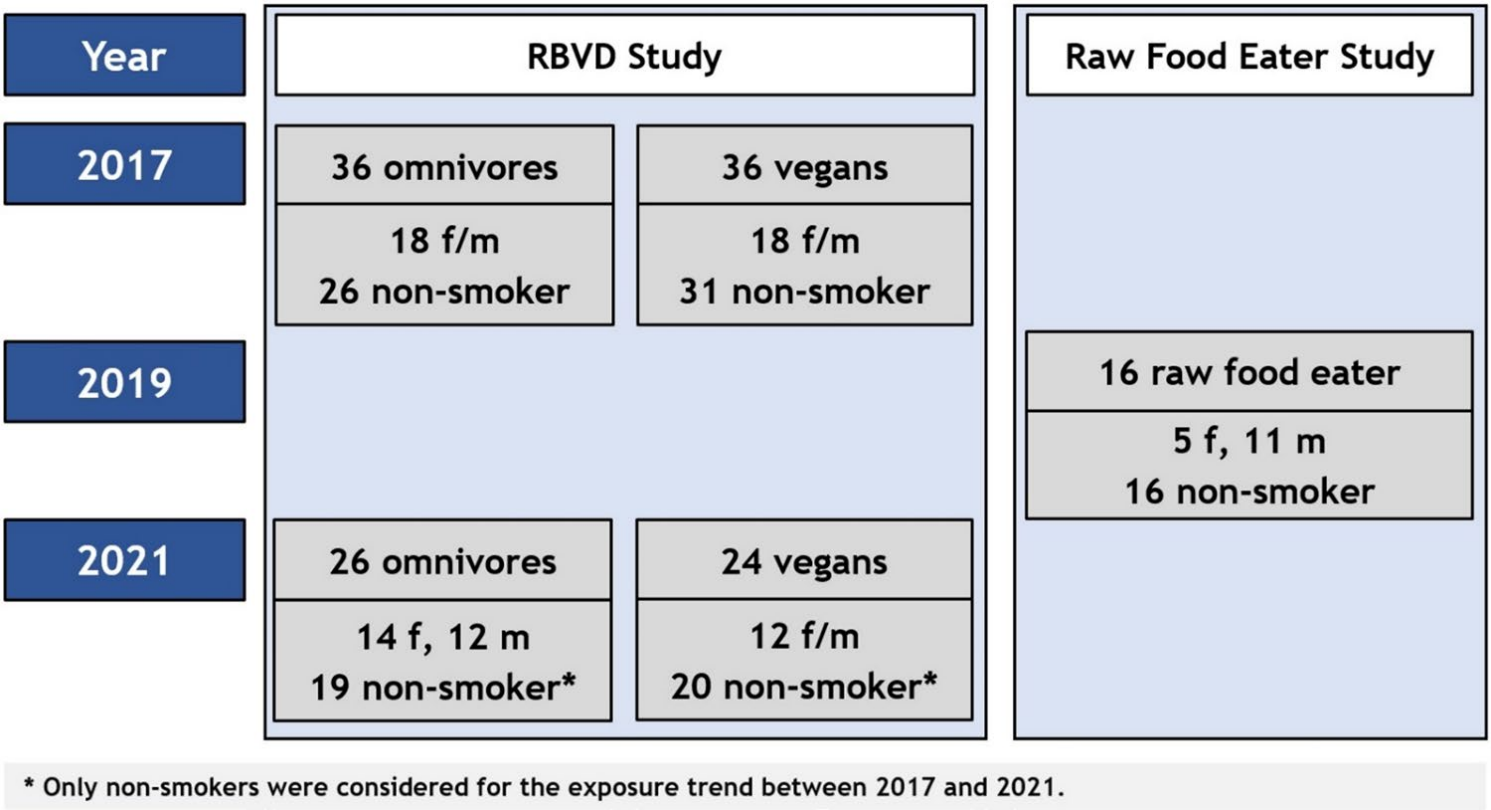
Blood and 24-h urine samples were from 88 adults examined in 2 different studies: The Risks and Benefits of a Vegan Diet (RBVD) study included 72 vegans and omnivores (Weikert et al. 2020), 50 of which were re-examined in 2021, and the Raw Food Eater Study included 16 participants (Abraham et al. 2022)

Samples of 24-h urine and blood were collected from 88 healthy adult study participants enrolled between 2017 and 2021 in two studies (Fig. 1). In the RBVD study, 36 vegans and 36 omnivores (sex- and age-matched, 18 females and 18 males in each group, 30 to 60 years old) were recruited in 2017 to compare the influence of the diet on nutrition and biomarker status. Omnivores were included, if they reported to consume at least three servings of meat or two servings of meat and two servings of sausages per week. Vegans did not eat any animal food. In both groups, the dietary habits had to have remained constant for at least 1 year (Weikert et al. 2020). From the 72 study participants examined in 2017, 24 vegans and 26 omnivores followed the invitation for a follow-up examination in 2021. For the assessment of the exposure time trend, only study participants that did not smoke in 2017 and in 2021 were considered (19 omnivores, 20 vegans) (Fig. 1).

The goal of the second cross-sectional study was the investigation of internal exposure to heat-induced food contaminants in people strictly abstaining from consumption of food heated to temperatures higher than 42 °C (raw food eater study) (Abraham et al. 2022). The study included 16 healthy subjects (11 males and 5 females, age between 20 and 65 years) following a raw food diet for at least four months (Fig. 1). Exclusion criteria were any consumption of beverages like coffee or tea or of hot meals and smoking.

The participants collected their urine completely for 24 h using preservative-free plastic containers provided during the first study visit, starting the day before the second study visit and ending with the first urine in the morning of the second visit. The samples were thoroughly mixed, weighed, aliquoted and stored at − 80 °C until analysis. Blood samples were taken using EDTA tubes S-Monovette^®^ (9 mL, Sarstedt, Numbrecht, Germany), centrifuged (2,500⋅*g*, 12 min) and the plasma was removed. The erythrocytes were washed twice with 0.9% aqueous sodium chloride (2.5 mL) and resuspended with 2.5 mL of water. The Hb content was determined with a HemoCue Hb 201 + analyzer (Radiometer, Willich, Germany) and the erythrocyte samples were stored at − 80 °C.

The RBVD study (no. EA4/121/16 and EA4/063/21) and the raw food eater study (No. EA4/040/19) were approved by the Ethics Committee of Charité University Medical Center Berlin. The raw food eater study (No. 00017436) and the RBVD follow-up study (No. 000 25857) were registered in the German Clinical Trials Register (DRKS). The studies were performed in accordance with the ethical standards laid down in the 1964 Declaration of Helsinki and its later amendments (Abraham et al. 2022; Weikert et al. 2020).

All participants got detailed oral consultations about the rationales and the methods of the studies and gave informed consent in writing after their first visit of the study center.

### Chemicals

Potassium hydrogen carbonate, aqueous ammonia (25%), phenylboronic acid (> 97%), sodium sulfate (anhydrous, granulated, for organic trace analysis), acetic acid, hydrochloric acid (13 N), diethyl ether, ethyl acetate, *tert*-butyl methyl ether (*t*BME), iso-hexane, iso-octane, methanol, and acetonitrile (for UHPLC-MS) were purchased from Merck KGaA (Darmstadt, Germany). Formic acid (≥ 96%), *N*,*N*-dimethylformamide (DMF), dimethyl sulfoxide (DMSO), and fluorescein-5-isothiocyanate (FITC, isomer I, > 95%) were provided by Sigma (Steinheim, Germany), and water (UHPLC MS-Optigrade) was supplied by LGC Standards GmbH (Wesel, Germany). Sodium sulfate was dried overnight in a muffle furnace (~ 200 °C) before use. All reagents and solvents were of analytical grade.

The isotope-labeled standard 3-chloro-1,2-propanediol-d_5_ (3-MCPD-d_5_) was obtained from Sigma-Aldrich (Steinheim, Germany) and 2-chloro-1,3-propanediol-d_5_ (2-MCPD-d_5_) was from LGC Standards GmbH. The quantification standard 3-(fluorescein-5-yl)-1-(2,3-dihydroxypropyl)-5-d_7_-isopropyl-2-thioxo-4-imidazolidinone (DHP-d_7_-Val-FTH) and the dipeptide *N*-(2,3-dihydroxypropyl)-Val-Leu-anilide (DHP-VL-An) were synthesized as described by Hielscher et al. (2017) and Abraham et al. (2019), respectively.

### Preparation of standard solutions

Amounts of ~ 1 mg of DHP-d_7_-Val-FTH or of the dipeptide DHP-VL-An were weighed and dissolved in respective volumes of DMSO to yield solutions of 5 mmol/L. The working solutions of DHP-d_7_-Val-FTH (50 nmol/L) or of the dipeptide DHP-VL-An (100 nmol/L) were prepared by dilution in water/acetonitrile (1:1), aliquoted for further use and stored at  − 80 °C.

Two separate stock solutions of 2-MCPD-d_5_ and 3-MCPD-d_5_ (1 g/L each) were prepared by dissolution of 10 mg 2-MCPD-d_5_ or 10 mg 3-MCPD-d_5_ in 10 mL methanol. The working solution (1 µg/L each of 2-MCPD-d_5_ and 3-MCPD-d_5_) was prepared by mixing 1 mL each of the stock solutions with 998 mL methanol. The working solution was aliquoted into 10 mL screw cap vials which were sealed with laboratory film and stored at room temperature.

### Edman degradation and solid-phase extraction (SPE) of DHP-Val-FTH

DHP-Val was cleaved from the N-termini of Hb using a modified Edman degradation with FITC as described (Fig. S1) (Gauch et al. 2022; Rydberg et al. 2009). Aliquots of erythrocyte samples containing 35 mg Hb were mixed with 15 µL of 1 M aqueous potassium hydrogen carbonate, 10 µL of the isotope-labeled standard solution containing DHP-d_7_-Val-FTH (50 nmol/L), and 5 mg FITC in 30 μL DMF. After incubating for 18 h at 37 °C, protein was precipitated by addition of 1.6 mL acetonitrile and the samples were centrifuged (18,000**⋅***g*, 10 min). The supernatant was pH-adjusted with 25 µL of 1 M aqueous ammonium hydroxide and transferred to an Oasis MAX cartridge (60 mg; Waters, Eschborn, Germany), preconditioned with 2 mL acetonitrile and 2 mL water. The cartridge was washed with 2 mL each of acetonitrile, water and 1% aqueous formic acid, and DHP-Val-FTH was eluted with 3 mL acetonitrile/water (9:1) containing 1% formic acid. The eluate was evaporated to dryness and reconstituted in 50 µL acetonitrile/water (1:1) containing 1% formic acid.

### UPLC–MS/MS analytical quantification of DHP-Val-FTH

The UPLC-MS system comprised an Acquity I-Class (Waters) connected to a triple quadrupole-hybrid ion trap mass spectrometer QTrap6500 (Sciex, Darmstadt, Germany) equipped with an electrospray ionization source operated in the positive mode. The DHP-Val-FTH (10 µL sample volume) was separated chromatographically using an Acquity HSS T3 column (1.8 µm, 2.1 mm × 150 mm, Waters). The eluent solvents were water + 0.1% formic acid (eluent A) and acetonitrile + 0.1% formic acid (eluent B). A two-step gradient was applied at a flow rate of 0.4 mL/min: 0–1 min (10% eluent B), 1–15 min (10–50% eluent B), 15–21 min (50–70% eluent B), 21–22.5 min (90% eluent B) and 22.5–24 min (10% eluent B). DHP-Val-FTH and DHP-d_7_-Val-FTH were recorded by multiple reaction monitoring (MRM). Table S1 in the Supplemental Information summarizes the fragmentation transitions and the respective parameters (declustering potential, entrance potential, collision energies and cell exit potentials). The other mass spectrometric parameters were set to the following values: curtain gas, 20 psi; ion source temperature, 450 °C; ion spray voltage, 5500 V; ion source gas 1, 60 psi; ion source gas 2, 50 psi; collision-activated dissociation gas, *medium*. The data were recorded and analyzed with Analyst 1.7.1 Software (Sciex).

The adduct levels were calculated using Eq. 1:

1 $${amount}_{adduct}\left[\frac{pmol}{g Hb}\right]=\frac{\frac{{A}_{analyte}}{{A}_{IS}} * {amount}_{IS} \left[pmol\right] }{{amount}_{Hb} \left[g\right]}$$

with A_analyte_ and A_IS_ as the peak areas of the quantifier signals of the analyte and of the internal standard, respectively, and with amount_IS_ and amount_Hb_ as the quantities of the internal standard and of Hb applied in the Edman degradation, respectively. The accuracy of quantification of DHP-Val was improved using ten control samples of an erythrocyte pool (incorporated in each sample set). Five of these samples were spiked with 10 µL of 100 nmol/L DHP-VL-An and otherwise worked up as described. These samples allowed determining the efficiency of the FITC-mediated Edman degradation, which was used to correct the DHP-Val levels determined in the human samples (Abraham et al. 2019; Monien et al. 2020). The limits of detection (LOD) and quantification (LOQ) were 0.25 and 0.71 pmol/g Hb. Further details on the validation for the determination of DHP-Val in Hb described previously (Gauch et al. 2022; Rydberg et al. 2009) are summarized in Table S2 of the Supplemental Information.

### Extraction and derivatization of 2/3-MCPD

Extraction from urine samples and derivatization of 2/3-MCPD using phenylboronic acid were described before (Abraham et al. 2021). Briefly, urine samples were thawed, mixed vigorously, and aliquots of 4 mL were spiked with 40 μL each of 3-MCPD-d_5_ (1 µg/L) and of 2-MCPD-d_5_ (1 µg/L). The samples were washed with 3.5 mL of *t*BME/isohexane (1:4). 2/3-MCPD were extracted using 2.5 mL of diethyl ether/ethyl acetate (9:1). Phase separation was completed by a 2-min centrifugation (3,500 rpm), and the extraction was repeated twice. The combined organic layers were dried over sodium sulfate, mixed with 50 μL of phenylboronic acid (2 mg/mL) in diethyl ether/ethyl acetate (9:1), and concentrated in a moderate nitrogen flow. A residual volume of 1 mL was dried with 200 mg sodium sulfate and the solutions were concentrated to dryness. The residuals were taken up in 200 μL of iso-octane, transferred into an analysis vial with a 200 µL-insert and stored in the freezer prior to GC–MS analysis. Analyses were carried out in duplicate.

### GC–MS analysis of 2/3-MCPD

The dioxaborolane derivatives of 2/3-MCPD (Fig. S2) were analyzed by an accredited laboratory (SGS Germany GmbH, Hamburg, Germany) using a gas chromatograph 7890B system (Agilent Technologies, Santa Clara, CA, USA) equipped with a Programmable Temperature Vaporizor (PTV) injection device. The following conditions were applied: injection volume 2–4 μL (pulsed splitless injection), PTV temperature program: 80 °C (isothermal for 0.1 min), increase with 2 °C/s to 140 °C (isothermal for 6 min), increase with 10 °C/s to 320 °C (isothermal for 10 min). PTV purge gas flow: 50 mL helium/min at 0.5 min to 1 min (septum purge 3 mL helium/min). Samples were separated on a Rxi-17 (Resteck GmbH, Bad Homburg, Germany) with a stationary phase of 50% diphenyl/50% dimethylpolysiloxane (30 m × 0.25 mm, 0.25 mm film thickness) and the pre-column HP-5 ms (Agilent Technologies) with a stationary phase of 5% phenyl/95% dimethylpolysiloxane (2.4 m × 0.32 mm, 0.25 mm film thickness). Helium was used as carrier gas at a constant flow rate of 1.4 mL/min. The oven temperature started at 80 °C (isothermal for 2 min) followed by a gradient of 5.4 °C/min up to 150 °C (isothermal for 4 min). Then, the temperature increased with 20 °C/min to 280 °C (isothermal for 5 min). Analytes were ionized by electron impact and detected using the single quadrupole mass spectrometer 5977B (Agilent Technologies) operated in the selected ion monitoring (SIM) mode. Mass-to-charge ratios (*m/z*) of the detected phenylboronic derivatives of the analytes were *m/z* = 146, 147, 196, 198 (3-MCPD), *m/z* = 149, 150, 201, 203 (3-MCPD-d_5_), *m/z* = 196, 198 (2-MCPD). 3-MCPD was quantified from the respective signal of the 3-MCPD-d_5_ phenyl boronic acid derivative using the ratio of the traces 147/150. 3-MCPD-d_5_ (*m/z* = 150) was also applied for the quantification of 2-MCPD (*m/z* = 196) with the help of a specific correction factor (2.47). Every eighth sample was a quality control sample (human urine with defined amounts of 2/3-MPCD). Ratios of the response factors, e.g., *m/z* = 150 vs. 147 (3-MCPD-d_5_ vs. 3-MCPD), were checked for constancy. The LOD and LOQ values were 0.1 and 0.25 μg/L for 3-MCPD, respectively, and 0.12 and 0.3 μg/L for 2-MCPD, respectively. Further validation data on 2/3-MCPD analyses in urine samples described by Abraham et al. (2021) are summarized in Table S3 of the Supplemental Information. The analytesʼ molecular structures are depicted in Supporting Information Fig. S1. Analyses were performed in duplicates.

### Reverse dosimetry estimations of daily intakes of 2/3-MCPD and glycidol

The daily oral intake of 2/3-MCPD (free or in form of fatty acid esters) was estimated from the excretion of 2/3-MCPD in 24-h urine samples using the ratio between the excreted amounts and the oral exposure (2-MCPD: 14.3 ± 3.1%; 3-MCPD: 3.73 ± 0.95%, mean ± SD) determined in 12 adult participants after the controlled exposure to a hazelnut oil with known high amounts of 2/3-MCPD fatty acid esters (Abraham et al. 2021). The glycidol intake was estimated from the DHP-Val levels. Assuming steady-state conditions for adduct formation after at least four months of continuous glycidol exposure, the daily exposure (*D*, µg/kg bw/day) can be calculated by Eq. 2,

2 $$D \left[\frac{\mu g}{kg\,bw\,d}\right]=\frac{2 * H \left[\frac{pmol}{g\,Hb}\right] }{\tau \left[d\right]*k\left[\frac{pmol}{g\,Hb}/\frac{\mu g}{kg\,bw}\right]}$$

with the adduct level (*H*), and the constants (*τ* = 104 d, mean lifetime of the adducts) and the mean adduct level/dose ratio (*k* = 0.082 ± 0.004 pmol DHP-Val/g Hb per μg glycidol/kg bw) determined in 11 adult participants after controlled exposure to glycidol fatty acid esters for 28 days (Abraham et al. 2019).

### Statistical analysis

Due to the influence of tobacco smoking on the exposure of 2/3-MCPD as well as glycidol, the data of smoking and non-smoking participants were evaluated separately. Daily urinary excretion of 2/3-MCPD and DHP-Val in Hb are reported as median values with the inter-quartile range (IQR) in brackets. Differences of biomarker levels were evaluated with the Mann–Whitney rank-sum test, and differences between biomarker levels determined in 2017 and 2021 were evaluated with a Wilcoxon signed rank test using SigmaPlot 14.0 (Systat Software, Inc., Erkrath, Germany). Differences with *p*-values < 0.05 were considered statistically significant. Spearman rank order correlations were calculated with SigmaPlot 14.0 (Systat Software, Inc.).

## Results

### Study populations

In the RBVD study, 36 vegans and 36 omnivores were enrolled, with 18 women in each group (Fig. 1). In 2017, the age range was between 30 and 57 years with average values of 38.5 and 37.5 years for omnivores and vegans, respectively. Vegan participants had been adhering to their diet for 1.6 to 20.2 years (median 4.8 years). Additional information can be found in Weikert et al. (2020). Thirteen participants identified themselves as smokers. Two E-cigarette users were added, resulting in a combined total of 15 smoking individuals (10 omnivores and 5 vegans).

The raw food eater study comprised 5 women and 11 men with an age between 23 and 63 years (mean 44.6 years). The diet had been followed for at least 4 months and up to 29 years (mean 11.6 years). A majority of the participants (56%, one woman and eight men) followed the diet for 10 years or more. The study participants had extensive knowledge on the processes in food production and specifically on possible heat treatments. The strong commitment was shown by the long duration of the habits, the willingness to accept daily difficulties to supply food (e.g., growing sprouts, shopping in specialized stores or via the internet) and to prepare meals, and the necessary abstinence from many foods as well as restaurant visits. The majority of participants relied on a diet primarily consisting of fresh fruits and raw vegetables, and eight participants emphasized the significance of nuts in their diets. They could be classified into three groups: raw vegans (*n* = 4), vegetarians (*n* = 4; occasional consumption of raw milk, cheese or eggs), and omnivores (*n* = 8; consumption of raw meat and/or raw fish). In the 3-day food records, the participants reported a rather limited selection of food groups compared to vegans and omnivores; only 23 out of 57 food groups were consumed by at least one of the participants. Consequently, certain food groups (such as fresh fruit, raw vegetables, raw milk, or meat) have been consumed in excessive quantities, occasionally surpassing 1 kg per day for 9 individuals. Additional information on the nutritional profile of the participants are provided by Abraham et al. (2022).

### Urinary excretion of 2-MCPD and 3-MCPD in omnivores, vegans and raw food eaters

The data were evaluated using all 2/3-MCPD signals with signal-to-noise (S/N) values between 3 (LOD) and 10 (LOQ) as such, because the validity of results is expected to be higher compared to that after replacement of the signals < LOQ with LOQ/2 (EFSA Working Group on Left Censored Data 2010). Both analytes were well detectable in the urine samples of the RBVD study with only four samples (2-MCPD) and one sample (3-MCPD) out of 122 samples with concentrations < LOD. However, in urine samples of raw food eaters, 2- and 3-MCPD were below the LOD in 13 and 11 samples, respectively. The 2/3-MCPD concentrations in urine samples with non-detects were set to LOD/2.

**Fig. 2:**
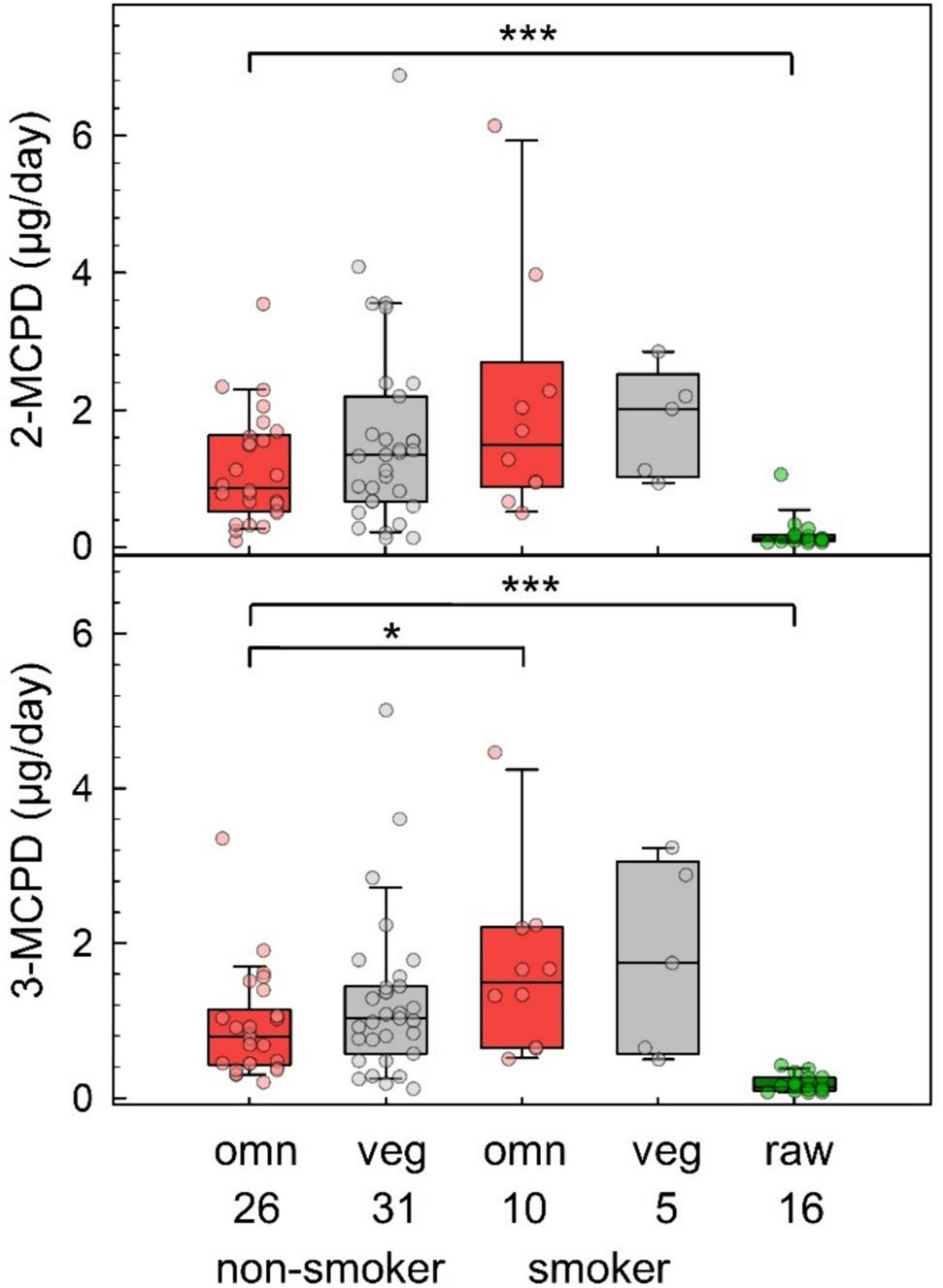
Daily excretion of 2-MCPD (upper panel) and 3-MCPD (lower panel) in non-smoking omnivores (red boxes, *n* = 26) and vegans (grey boxes, *n* = 31) from the RBVD study in 2017 (left columns), in the subgroups of smoking omnivores (red boxes, *n* = 10) and vegans (grey boxes, *n* = 5, middle columns), and in strict raw food eaters (green boxes, *n* = 16, right columns). Boxes represent lower and upper quartiles and middle lines the median values. The error bars represent the 10th and 90th percentiles. Significant differences are noted with asterisks (*p ≤ 0.05, ***p ≤ 0.001, Mann–Whitney rank-sum test)

The daily excretion of 2/3-MCPD in non-smoking and smoking omnivore and vegan study participants of the RBVD study (2017) in comparison to that observed for raw food eaters is depicted in Fig. 2. The difference between 2-MCPD excretion of 0.87 µg/day for omnivores and of 1.35 µg/day for vegans (Fig. 2, upper panel) was not statistically significant (*p* = 0.33). The median urinary excretion of 2-MCPD in smoking omnivores (1.49 µg/day) and smoking vegans (2.02 µg/day) were not significantly different if compared to the excretion of non-smoking omnivores (*p* = 0.12) and vegans (*p* = 0.34) (Table 1). The median levels of 3-MCPD excretion were 0.79 and 1.03 µg/day for omnivores and vegans, respectively (Table 1). The difference was not statistically significant (*p* = 0.20). Median values of 3-MCPD excretion for smokers were 1.49 and 1.74 µg/day for omnivores and vegans, respectively. The differences of 3-MCPD excretion observed for non-smokers and smokers were significant in omnivores (*p* = 0.025) but not in vegans (*p* = 0.36).

**Table 1:** Minima, 25%, median, 75% and maxima of urinary daily 2/3-MCPD excretion and exposure estimates of smoking and non-smoking omnivores and vegans as well as strict raw food eaters

	n	min	0.25%	median	0.75%	max	median intake estimate^*a*^
		µg/day	µg/day	µg/day	µg/day	µg/day	µg/kg bw/day
2-MCPD							
Omnivores/non-smokers	26	0.10	0.52	0.87	1.63	3.54	0.09
Vegans/non-smokers	31	0.14	0.66	1.35	2.20	6.88	0.12
Omnivores/smokers	10	0.50	0.87	1.49	2.70	6.14	(0.16)^*b*^
Vegans/smokers	5	0.94	1.03	2.02	2.53	2.85	(0.19)^*b*^
Raw food eaters	16	0.06	0.09	0.13	0.18	1.06	(0.02)^*c*^
3-MCPD							
Omnivores/non-smokers	26	0.20	0.42	0.79	1.14	3.35	0.30
Vegans/non-smokers	31	0.12	0.57	1.03	1.44	5.01	0.38
Omnivores/smokers	10	0.50	0.65	1.49	2.20	4.46	(0.59)^*b*^
Vegans/smokers	5	0.50	0.57	1.74	3.06	3.23	(0.63)^*b*^
Raw food eaters	16	0.06	0.09	0.14	0.26	0.42	(0.06)^*c*^

^a^The median daily oral intake of 2/3-MCPD was estimated from the individual 2/3-MCPD excretion using the mean conversion factors for 2-MCPD (14.3%) and 3-MCPD (3.73%) (Abraham et al. 2021)

^b^The conversion factors applied for the estimation were calculated after oral exposure to a 2/3-MCPD ester-rich hazel nut oil, and thus, may not apply to the inhalative uptake of 2/3-MCPD via tobacco smoke

^c^Most urine samples of raw food eaters did not contain detectable amounts of 2/3-MCPD. The replacement of these values with LOD/2 results in a possible overestimation of the intake estimates

Due to multiple replacements of low signals (S/N < 3) with LOD/2 values, the median excretion values for 2- and 3-MCPD observed in raw food eaters, 0.13 µg/day and 0.14 µg/day, respectively, must be considered as rough estimates. The amounts were significantly lower compared to 2/3-MCPD excretions observed in non-smoking omnivores and vegans (*p* ≤ 0.001 each).

### N-(2,3-Dihydroxypropyl)-Val (DHP-Val) in Hb of omnivores, vegans and raw food eaters

Analyzing blood samples of the RBVD study, all DHP-Val signals (122 samples measured in duplicates) were well above the LOD, however, 19 samples had signals between the LOD and the LOQ. In the samples of raw food eaters, all of the signals (16 samples measured in duplicates) were higher than the LOD, but only two signals exceeded the LOQ. As recommended, the DHP-Val levels between the LOD and the LOQ were used as such for the evaluation (EFSA Working Group on Left Censored Data 2010).

**Fig. 3:**
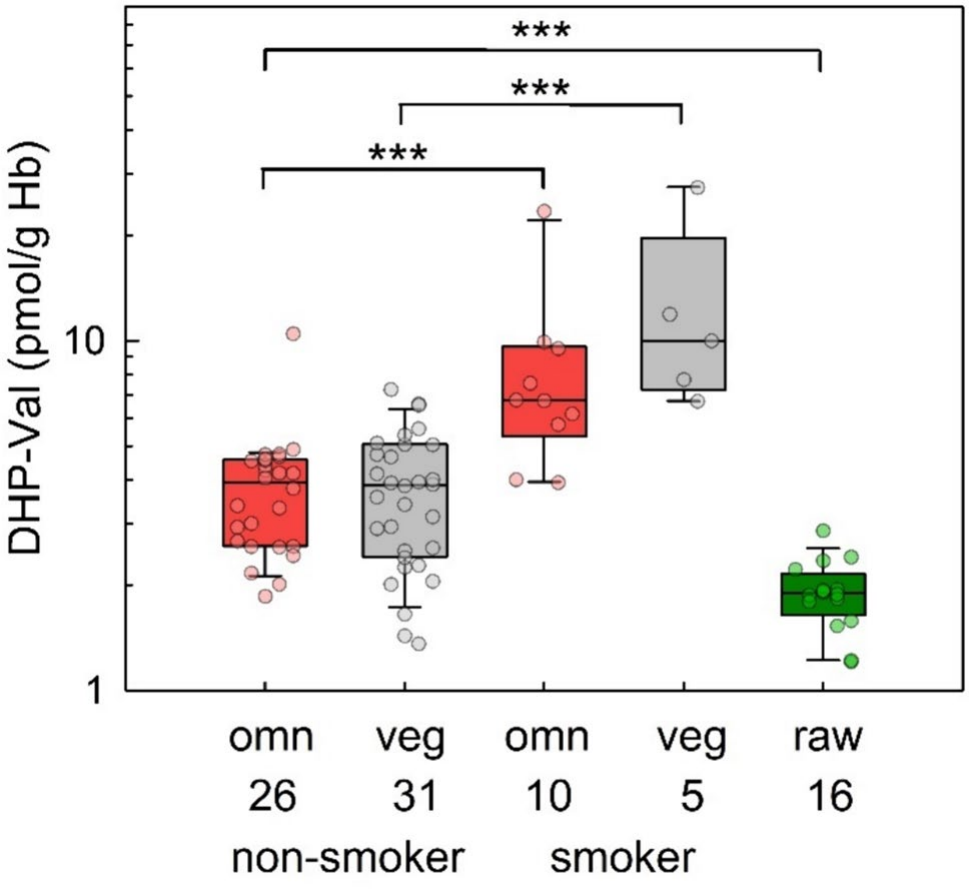
Levels of DHP-Val in non-smoking omnivore (red box, *n* = 26) and vegan (grey box, *n* = 31) adult study participants from the RBVD study in 2017 (left columns), in smoking omnivores (red box, *n* = 10) and vegans (grey box, *n* = 5, middle columns), and in strict raw food eaters (green box, *n* = 16, right column). Boxes represent lower and upper quartiles and middle lines the median values. The error bars represent the 10th and 90th percentiles. Significant differences are noted with asterisks (***p ≤ 0.001, Mann–Whitney rank-sum test)

DHP-Val levels in blood samples from omnivores and vegans at the first time of the examination and of the participants of the raw food eater study are shown in Fig. 3. Blood samples of non-smoking omnivores and vegans of the RBVD study contained median DHP-Val levels of 3.9 pmol/g Hb each (Table 2). In the group of smokers, median DHP-Val levels in omnivores and vegans were 6.8 pmol/g Hb and 10.0 pmol/g Hb, respectively. The differences of DHP-Val in Hb between smokers and non-smokers were statistically significant in omnivores and vegans (*p* < 0.001 each). The DHP-Val level in blood samples from raw food eaters (median 1.9 pmol/g Hb) were significantly lower compare to those of omnivores and vegans (*p* < 0.001 each).

**Table 2:** Minima, 25%, median, 75% and maxima of DHP-Val in Hb samples and estimated glycidol exposure of smoking and non-smoking omnivores and vegans as well as strict raw food eaters

	n	min	25%	median	75%	max	median intake estimate^*a*^
		pmol/g Hb	pmol/g Hb	pmol/g Hb	pmol/g Hb	pmol/g Hb	µg/kg bw/day
Omnivores/non-smokers	26	1.9	2.6	3.9	4.6	10.5	0.92
Vegans/non-smokers	31	1.4	2.4	3.9	5.1	7.3	0.90
Omnivores/smokers	10	3.9	5.3	6.8	9.6	23.5	(1.59)^*b*^
Vegans/smokers	5	6.7	7.2	10.0	19.7	27.5	(2.35)^*b*^
Raw food eaters	16	1.2	1.6	1.9	2.2	2.9	(0.44)^*b*^

^a^The median exposure was calculated (Eq. 2 in the method section) from individual DHP-Val levels using the mean adduct level/dose ratio (*k* = 0.082 ± 0.004 pmol DHP-Val/g Hb per μg glycidol/kg bw) (Abraham et al. 2019)

^b^Due to the suspected differences of toxicokinetic fates of glycidol inhaled via tobacco smoke or consumed orally (in form of fatty acid esters), the estimations does not strictly apply to the inhalative exposure in smokers or to the exposure of unknown origin in raw food eaters

### Estimation of daily uptake of 2-MCPD, 3-MCPD and glycidol by reverse dosimetry

The calculation of the exposure to 2/3-MCPD was restricted to non-smokers, because the conversion factors for the oral dietary uptake of MCPD esters and the inhalation of free MCPDs in tobacco smoke may be different. Assuming that 2/3-MCPD exposure was exclusively due to dietary sources, the excreted amounts in non-smoking omnivores and vegans corresponded to median dietary intakes of 0.09 and 0.12 µg/kg bw/day 2-MCPD, respectively, and of 0.30 and 0.38 µg/kg bw/day 3-MCPD, respectively (Table 1). In the case of raw food eaters, the estimation of 2/3-MCPD in urine samples by replacement of most non-detects with LOD/2 did not allow approximating the oral exposure with confidence.

The daily oral intake of glycidol was estimated from DHP-Val levels using the conversion factor by Abraham et al. (2019). The DHP-Val levels determined in the current study allowed to estimate a median daily glycidol exposure of 0.92 µg/kg bw/day (omnivores), 0.90 µg/kg bw/day (vegans) and 0.44 µg/kg bw/day (raw food eaters; Table 2). The latter value must be regarded as uncertain. The sources of exposure (inhalative, dermal or endogenous) in raw food eaters are unknown, and due to the suspected differences of toxicokinetic fates of glycidyl esters taken up orally or glycidol exposure by other routes, the estimation must not apply to the DHP-Val levels of raw food eaters.

### Urinary excretion of 2/3-MCPD and DHP-Val in Hb of omnivores and vegans in 2017 and 2021

**Fig. 4:**
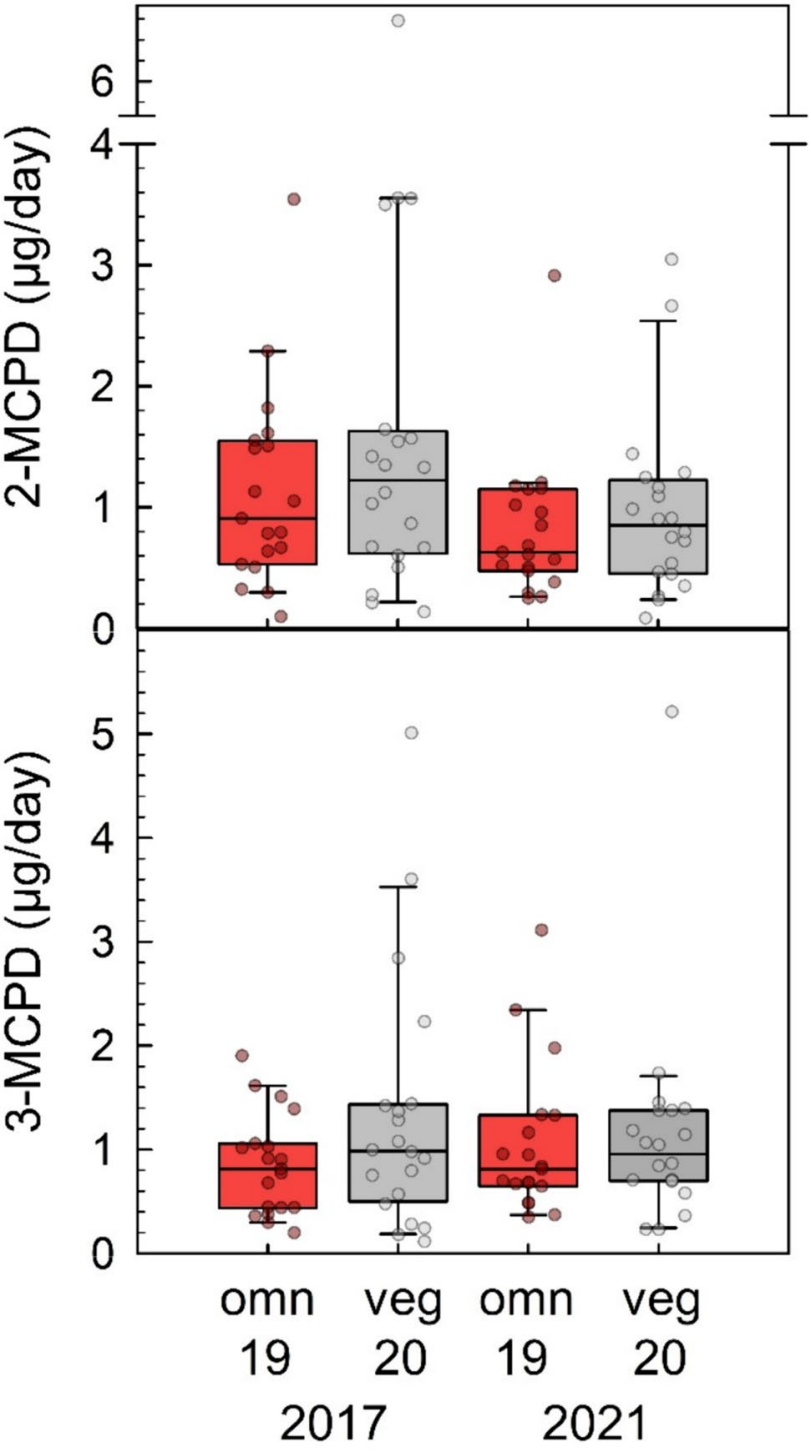
Daily urinary excretion of 2-MCPD (upper panel) and 3-MCPD (lower panel) in non-smoking omnivore (red boxes, *n* = 19) and vegan (grey boxes, *n* = 20) study participants in 2017 (left columns) and in 2021 (right columns). Lines and boxes represent median values and the lower and upper quartiles, respectively, and the error bars represent the 10th and 90th percentiles. The daily 2/3-MCPD excretion in 2017 in omnivores and vegans was not significantly different from those determined in 2021 (Wilcoxon signed-rank test). However, there was a significant decrease of 2-MCPD excretion from 2017 to 2021 considering omnivores and vegans combined (*p* = 0.02)

The time trends of exposure were compared at hand of non-smoking participants that presented themselves 4 years after the first examination for a second time (19 omnivores and 20 vegans). In 2017, the daily 2-MCPD excretion of omnivores and vegans was 0.91 (IQR 0.53, 1.55) µg/day and 1.22 (IQR 0.62, 1.63) µg/day, respectively, and in 2021, 0.63 (IQR 0.48, 1.15) µg/day and 0.85 (IQR 0.45, 1.23) µg/day, respectively (Fig. 4). The decreases with time were not significant (*p* = 0.11 each). However, if omnivores and vegans were considered as a single group, the difference was significant (*p* = 0.02). In the case of 3-MCPD, the median daily excretion of omnivores and vegans was 0.81 (IQR 0.44, 1.06) µg/day and 0.99 (IQR 0.50, 1.44) µg/day, respectively. In 2021, the median amounts of excreted 3-MCPD of omnivores and vegans were 0.81 (IQR 0.65, 1.33) µg/day and 0.96 (IQR 0.70, 1.38) µg/day, respectively. The difference between the two time points was not statistically significant (omnivores: *p* = 0.60, vegans: *p* = 0.84).

**Fig. 5:**
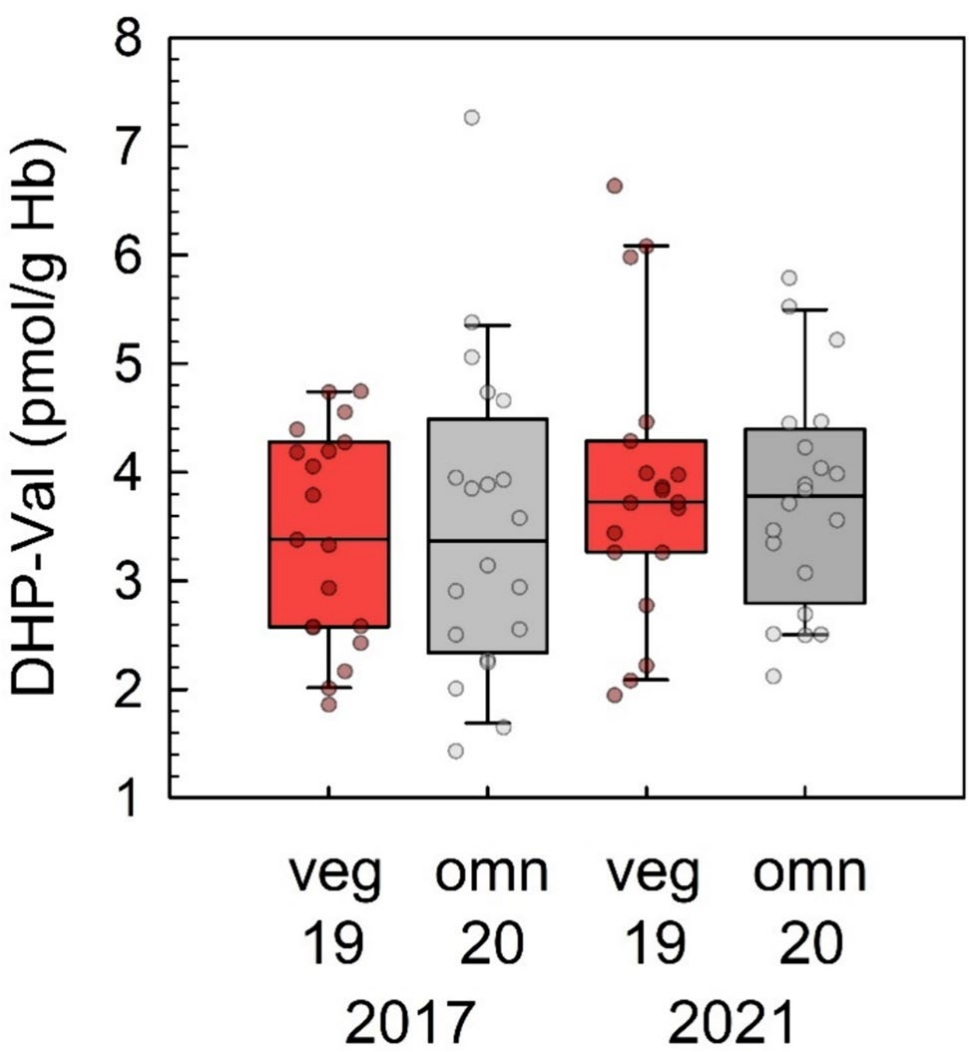
DHP-Val levels in non-smoking omnivore (red boxes, *n* = 19) and vegan (grey boxes, *n* = 20) study participants in 2017 (left columns) and in 2021 (right columns). Lines and boxes represent median values and the lower and upper quartiles, respectively, and the error bars represent the 10th and 90th percentiles. DHP-Val levels in 2017 in omnivores and vegans were not significantly different from those determined in 2021 (Wilcoxon signed-rank test). There was a small and significant increase of DHP-Val from 2017 to 2021 considering omnivores and vegans combined (*p* = 0.047)

The data on DHP-Val in blood samples of non-smoking omnivores and vegans that were examined twice are depicted in Fig. 5. In 2017, median DHP-Val levels in omnivores and vegans were 3.4 (IQR 2.6, 4.3) pmol/g Hb and 3.4 (IQR 2.3, 4.5) pmol/g Hb, respectively. In 2021, the median DHP-Val levels in the same omnivore and vegan participants were 3.7 (IQR 3.3, 4.3) pmol/g Hb and 3.8 (IQR 2.8, 4.4) pmol/g Hb, respectively. The differences between the adduct levels at both time points were not statistically significant (omnivores: *p* = 0.23, vegans: *p* = 0.076).

### Correlations between the two measurements (2017 and 2021) of urinary 2/3-MCPD and DHP-Val in Hb

**Fig. 6:**
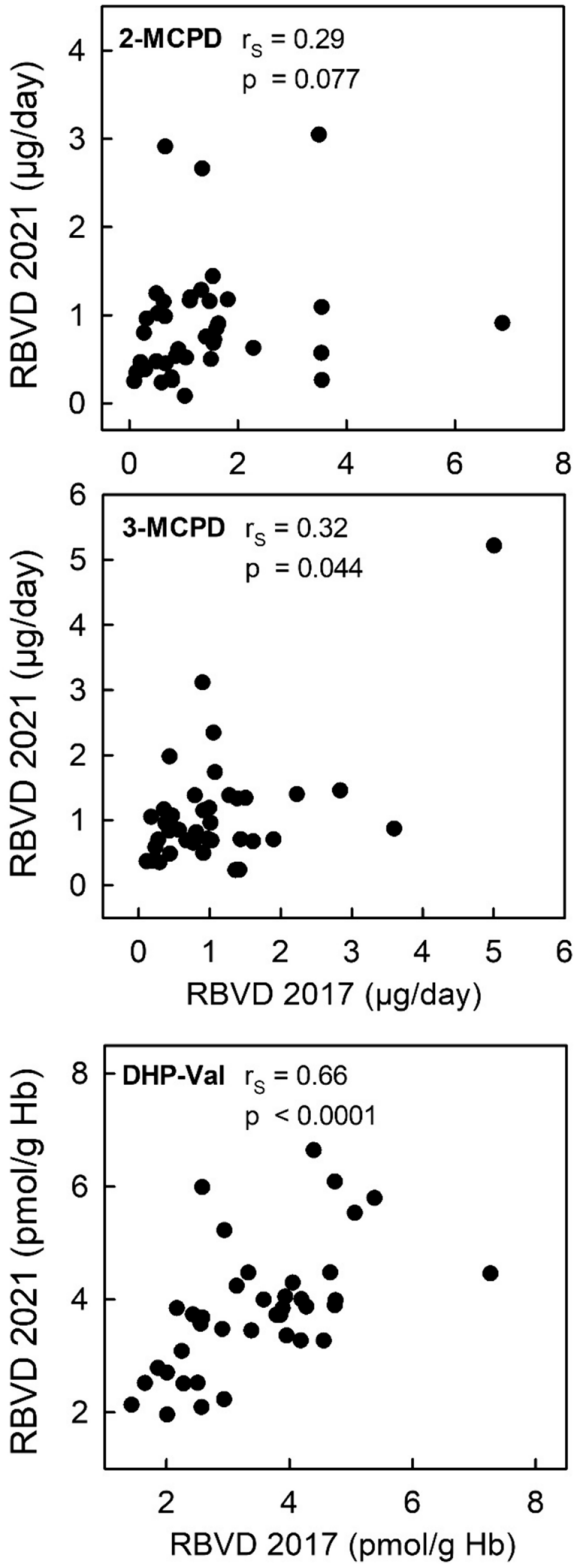
Scatterplots for the biomarkers determined in samples from 2017 (abscissae) and from 2021 (ordinates) in non-smoking participants (*n* = 39) of the RBVD study: urinary excretion of 2-MCPD (upper panel), of 3-MCPD (middle panel), and DHP-Val in Hb (lower panel)

Figure 6 shows scatterplots of the daily 2/3-MCPD excretion as well as DHP-Val levels in Hb determined in non-smoking participants of the RBVD study examined in 2017 and in 2021 (19 omnivores, 20 vegans). There was no correlation between 2-MCPD excretion at both time points (*r*_S_ = 0.29, *p* = 0.077) and a weak correlation between the excreted amounts of 3-MCPD (*r*_S_ = 0.32, *p* = 0.044). In contrast, there is a positive moderate correlation between DHP-Val levels measured four years apart (*r*_S_ = 0.66, *p* < 0.0001). The stability of individual levels over time can also be determined using intraclass correlation coefficients (ICC), which were low for 2-MCPD and 3-MCPD (0.13 each), and moderate 0.56 for DHP-Val (according to the classification of (Koo and Li 2016)).

## Discussion

In recent decades, the increasing health awareness and ethical responsibility towards animal welfare brought about alternative diets, characterized by evasion of meat and dairy products or heated foods. Human studies were conducted on associations between vegan diets and health, e.g., by analyzing vitamin- and nutrient levels in blood or urine (Weikert et al. 2020), or by monitoring the internal exposure to contaminants, e.g., mycotoxins (Fleury et al. 2017; Penczynski et al. 2022), perfluoroalkyl substances (PFAS) (Menzel et al. 2021), or heat-induced contaminants (Goerke et al. 2019). To the latter topic, we dedicate a series of articles entitled "*Internal Exposure to Heat-induced Food Contaminants in Omnivores, Vegans and Strict Raw Food Eaters*" (Monien et al. 2024). In these studies, biomarkers are used to determine the internal exposure, in order to avoid error-prone estimations of the external exposure and to include all possible contributing sources. Here, we addressed the question of the association between dietary habits and exposure to three heat-related contaminants with C_3_-backbones: 2-MCPD, 3-MCPD and glycidol.

### Exposure to 2/3-MCPD and glycidol in vegans, omnivores and raw food eaters

The daily urinary excretion of 2/3-MCPD was somewhat higher in vegans than in omnivores (Table 1), the differences, however, were not significant. These levels in the present study are consistent with the average daily urinary excretion of 2-MCPD (1.26 μg) and 3-MCPD (0.85 µg) observed in 12 German adults in 2017 (Abraham et al. 2021), which is the only other study reporting the human urinary 2/3-MCPD excretion as yet. In urine samples of raw food eaters, the concentrations were mostly below the LOD. This supports that there is indeed hardly any dietary 2/3-MCPD exposure of raw food eaters and that, in contrast to acrylamide (Monien et al. 2024) or acrolein (Ruenz et al. 2019), the monochloropropanediols may not be taken up otherwise in considerable amounts or formed endogenously.

A biomarker of glycidol exposure is the Hb adduct DHP-Val, which reflects the intake over a few months. The median values of DHP-Val in blood samples of vegans and omnivores (Table 2) indicated that there was no difference of glycidol exposure due to these dietary habits. The current data were comparable to those of previous studies reporting DHP-Val levels in non-smoking adults of 6.2 pmol/g globin (median, *n* = 42) (Honda et al. 2012), 4.0 pmol/g Hb (mean, *n* = 11) (Abraham et al. 2019), and 5.4 pmol/g Hb (median value of mothers at delivery, *n* = 85) (Monien et al. 2020). The highest mean level (10.3 pmol/g Hb) reported by Aasa et al. (2019b) was determined from six blood samples collected from adult study participants in 1997. It was surprising that raw food eaters showed detectable background levels of DHP-Val in Hb, corresponding to about 50% of those observed for vegans and omnivores. There are various possible explanations. Firstly, according to a hypothesis of Honda et al. (2014), DHP-Val may not be specific for the external exposure to glycidol. However, there is no literature on reactive metabolites of xenobiotics other than glycidol that form the adduct DHP-Val in relevant amounts. Neither one of the previously suspected 3-MCPD (Aasa et al. 2017a) and epichlorohydrin (Landin et al. 1997) did contribute significantly to DHP-Val in animal experiments. In two current studies, Shimamura et al. (2024b) showed that DHP-Val was elevated in mice treated with oral doses of glycidol and glycidol oleate, but not with the putative alternative precursors, propylene oxide, 1-bromopropane, allyl alcohol, fructose, or acrylic acid and 1,2-propanediol. Repeated doses of 3-MCPD, epichlorohydrine and glyceraldehyde increased DHP-Val, however, the adduct levels were over 100 times smaller than those observed after equimolar doses of glycidol (Shimamura et al. 2024b). Secondly, unidentified sources of glycidol in food (but also apart from the diet) may contribute to the external exposure that affect also raw food eaters. For example, an inhalative exposure cannot be completely ruled out. However, the (non-smoking) participants of the raw food eater study denied to be in contact with smokers of cigarettes, other tobacco products or e-cigarettes, and there are no data on the presence of glycidol as air pollutant in the domestic or urban environment. Thirdly, DHP-Val may be formed by glycidol from endogenous sources (Monien and Abraham 2023), or from reactions of the N-terminal Val in Hb with similar natural C_3_-compounds. Aasa et al. (2019b) suggested that glyceraldehyde and glycidaldehyde may contribute to the formation of DHP-Val. However, this requires the reduction of the intermediate imines formed from *N*-terminal amine of Hb and the aldehydes, a reaction that is not supposed to happen under physiological conditions. Considering the possibility of an endogenous exposure it is of note, that the database on alternative sources of exposure (dietary and non-dietary, e.g., through the environment or consumer products) is not as broad for glycidol as it is, for example, for acrylamide. Thus, the hypothesis of endogenous formation of glycidol may not be as likely as in the case of acrylamide, in which alternative sources can be practically ruled out (Monien et al. 2024).

### Free 2/3-MCPD and glycidol in cigarette smoke or e-cigarette aerosols

It has been known for long that cigarette smoke contains heat-induced contaminants like acrylamide (Diekmann et al. 2008), and previous biomarker studies confirmed its higher exposure in smokers (Urban et al. 2006). In contrast, the detection of 3-MCPD in tobacco smoke (Bentley et al. 2020) but also in e-cigarette aerosols (St Helen et al. 2018), probably as a product of heating sucralose in the e-liquids (El-Hage et al. 2019) or of the additive glycerol in *heated tobacco products* (HTPs) (Lang et al. 2024), are relatively recent findings, and there are hardly any quantitative data that allow assessing the ratio of inhalative to dietary exposure. Here, we showed for the first time by urine biomonitoring that there is a tendency towards a higher exposure to 2/3-MCPD in smokers compared to non-smokers (Table 1, Fig. 2); however, only the difference of 3-MCPD excretion in smoking vs. non-smoking omnivores was statistically significant. Due to the small number of study participants and lacking knowledge on toxicokinetic differences, the data do not allow comparing (quantitatively) dietary and inhalative exposure.

Glycidol occurs in cigarette smoke due to the formation from dehydration of glycerol (Lang et al. 2024; Sleiman et al. 2016), which is added to tobacco to improve moisture characteristics, and to support the application of flavors (Carmines and Gaworski 2005). The median levels of DHP-Val in vegan and omnivore smokers were 1.7- and 2.5-fold higher, respectively, compared to those of non-smokers (Table 2). This is in rough agreement with previous studies. In blood samples of non-smoking and smoking adults from 1997 (*n* = 6 each), Aasa et al. (2019a) observed mean DHP-Val levels of 10.3 and 23.4 pmol/g Hb, respectively. In blood samples of non-smoking (*n* = 85) and smoking mothers at delivery (*n* = 6), the median DHP-Val levels were 5.4 and 16.7 pmol/g Hb, respectively (Monien et al. 2020). As mentioned for urinary 3-MCPD excretion, DHP-Val levels in non-smokers and smokers should be compared with caution, because the toxicokinetic fates of bound glycidol after oral uptake and free glycidol after inhalation or endogenous formation are expected to be different.

### Time trends of exposures to 2/3-MCPD and glycidol

Maximum levels for 3-MCPD and glycidol (free and bound as fatty acid esters) have been established in the EU (European Commission 2023), and the food industry has been working on the mitigation by optimizing the refinement processes of plant oils (Oey et al. 2019; Yung et al. 2023). In order to verify the effectiveness of such measures, human studies on 2/3-MCPD and glycidol exposure at regular time intervals can be helpful. Our data did not show exposure changes for 3-MCPD (Fig. 4) and glycidol (Fig. 5) between 2017 and 2021 in the RBVD study, but a decrease of 2-MCPD exposure (Fig. 4), which was significant if all 39 adults were considered as a single group. The missing decrease of internal exposure to 3-MCPD and glycidol in this time frame despite mitigation strategies and EU regulation may be considered surprising, however, representative occurrence data of the compounds in foods on the German market are not available for comparison.

### Estimating the daily exposure to 2/3-MCPD and glycidol

Free 2- and 3-MCPD were hardly detectable in 24-h urine samples of raw food eaters, indicating that the diet of raw food eaters is indeed devoid of the corresponding fatty acid esters, and that 2- or 3-MCPD are not formed in significant amounts endogenously or from metabolism of other xenobiotics. Thus, the biomarkers 2/3-MCPD are relatively specific, which allows the estimation of the daily intake (in free form or as fatty acid esters) from the urinary excretion (Table 1). The estimated median levels of the 2-MCPD intake of non-smoking omnivores (0.09 µg/kg bw/day) and vegans (0.12 µg/kg bw/day) are comparable to the EFSA estimates for European adults, with a median value for the mean chronic exposure to 2-MCPD of 0.1–0.2 μg/kg bw/day. Also, the median 3-MCPD exposure (omnivores 0.30 µg/kg bw/day, vegans 0.38 µg/kg bw/day) calculated from the urinary excretion was similar to the estimate of the mean chronic exposure to 3-MCPD in adults of the European population (0.3 μg/kg bw/day) by EFSA (2016). In 2020, the German Federal Institute for Risk Assessment (BfR) estimated median total intakes for 2- and 3-MCPD to be 0.1 and 0.1–0.2 µg/kg bw, respectively, using food consumption data (obtained in 2005/2006; *n* = 15,371 adults) from the German National Nutrition Survey II as a basis (German Federal Institute for Risk Assessment 2020).

Assuming the specificity of the biomarker (as discussed above) and a steady-state exposure over several months, the DHP-Val levels allowed to estimate the daily glycidol intake of the study participants (Table 2). The estimates for non-smoking omnivores and vegans were nearly five times higher than the mean chronic exposure to glycidol for the adult population determined from dietary surveys and occurrence data by the EFSA (0.2 μg/kg bw) (EFSA 2016). The difference is even greater considering the estimates from the BfR for the median glycidol intake in German adults (0.1 μg/kg bw) in 2020 (German Federal Institute for Risk Assessment 2020). Possible reasons for these differences, three of which (alternative compounds forming DHP-Val, alternative sources or endogenous exposure to glycidol) were mentioned above, affect omnivores, vegans and raw food eaters in the same manner. These may explain the DHP-Val observed in raw food eaters and up to 50% of the level observed in non-smoking omnivores and vegans. However, the other half of the DHP-Val in omnivores, corresponding to an estimated exposure of about 0.45 µg glycidol/kg bw, is characteristic for the difference of dietary preferences between raw food eaters and omnivores. This value is about 2 to 4.5-fold higher compared to the dietary exposure estimates of EFSA and BfR. However, exposure data of different populations (and different times) are difficult to compare, also because there are weaknesses in both approaches. The numbers of subjects in the current human studies are relatively small. Furthermore, the exposure estimates of EFSA and BfR may be too low. Yet unidentified foods possibly add relevant levels to the overall exposure, and the glycidol formation by domestic food preparation procedures, like grilling meat (Inagaki et al. 2016; Shimamura et al. 2024a), or baking biscuits and cakes (Shimamura et al. 2024a), under household conditions was not considered.

## Conclusion

The absence of quantifiable amounts of 2/3-MCPD in the urine of raw foodists confirmed the specificity for the dietary exposure, allowing the application for reverse dosimetry. However, the compounds have a relatively short half-life in humans (after consumption of the respective esters from hazelnut oil: 2-MCPD: 5.4 h, 3-MCPD: 3.5 h (Abraham et al. 2021)). Thus, the urinary 24-h excretions are only suitable as biomarkers of short-term exposure, and, in agreement, the correlation between individual amounts determined in 2017 and 2021 is poor (Fig. 6). In case of DHP-Val, it is the other way round. The correlation of the DHP-Val levels between 2017 and 2021 indicated the high performance of the long-term exposure marker as well as a high consistency of dietary and life-style habits by the study subjects.

Both biomarkers can be used in human studies to monitor the temporal course of exposure to 2/3-MCPD and glycidol and to prove the effectiveness of regulation and mitigation measures. The question of alternative sources of glycidol exposure (including endogenous formation) or other reasons, why DHP-Val is detected independently of oral intake of heat-induced food, should be addressed by future research. If its specificity is clarified, and if the source is endogenous formation leading to a roughly doubling of the internal exposure from dietary exposure to glycidol, the related risk would be greater than that resulting from dietary uptake alone.

## Supplementary Information

Below is the link to the electronic supplementary material.

Supplementary file1 (DOCX 167 KB)

## Data Availability

The datasets generated during and/or analysed during the current study are not publicly available due to a lack of consent from study participants but are available from the corresponding author on reasonable request.

## Abbreviations

BfR: German Federal Institute for Risk Assessment
bw: Body weight
DHP-Val: *N*-(2,3-Dihydroxypropyl)-Val
DHP-Val-FTH: *N*-(2,3-Dihydroxypropyl)-Val fluorescein thiohydantoin
EFSA: European Food Safety Authority
EU: European Union
FITC: Fluorescein-5-isothiocyanate
FTH: Fluorescein thiohydantoin
Hb: Hemoglobin
LOD: Limit of detection
LOQ: Limit of quantification
2-MCPD: 2-Monochloro-1,3-propanediol
3-MCPD: 3-Monochloro-1,2-propanediol
MRM: Multiple reaction monitoring
RBVD study: Risks and benefits of a vegan diet study
S/N: Signal-to-noise ratio
SPE: Solid-phase extraction
UPLC-MS/MS: Ultra-performance liquid chromatography–tandem mass spectrometry
