# Supplementary material for: Internal exposure to heat-induced food contaminants in omnivores, vegans and strict raw food eaters: biomarkers of exposure to 2- and 3-monochloropropanediol (urinary excretion) and glycidol (hemoglobin adduct N-2,3-dihydroxypropyl-Val)
Source: Arch Toxicol. 2024 Oct 1;99(1):271–85. doi: 10.1007/s00204-024-03880-6 (PMC11742272; doi:10.1007/s00204-024-03880-6)
Supplement: Supplementary file 1 — Supplementary file1 (DOCX 167 KB) [file 204_2024_3880_MOESM1_ESM.docx]

**Internal Exposure to Heat-induced Food Contaminants in Omnivores, Vegans and Strict Raw Food Eaters: Biomarkers of Exposure to 2- and 3-Monochloropropanediol (Urinary Excretion) and Glycidol (Hemoglobin Adduct *N*-2,3-Dihydroxypropyl-Val)**

Bernhard H. Monien, Jan Kuhlmann, Fabian Gauch, Cornelia Weikert, Klaus Abraham

**Supplemental Information**


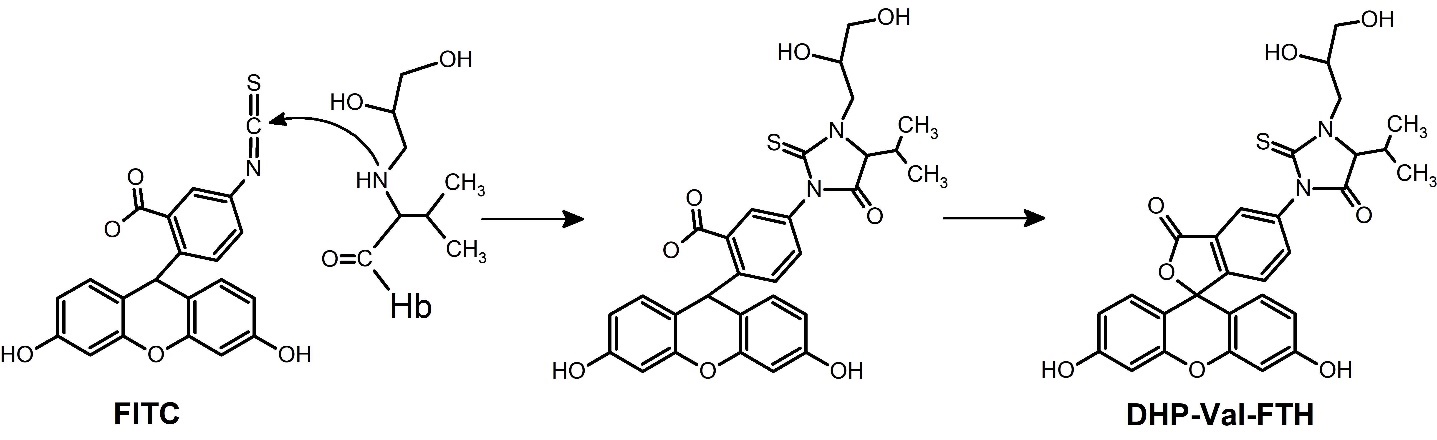


**Figure S1** Modified Edman degradation of DHP-Val with the FIRE procedure™. The adduct DHP-Val in Hb is cleaved using fluorescein-5-isothiocyanate (FITC) to yield *N*-(2,3-dihydroxypropyl)-valine fluorescein thiohydantoin (DHP-Val-FTH), which is quantified by isotope-dilution UHPLC-MS/MS


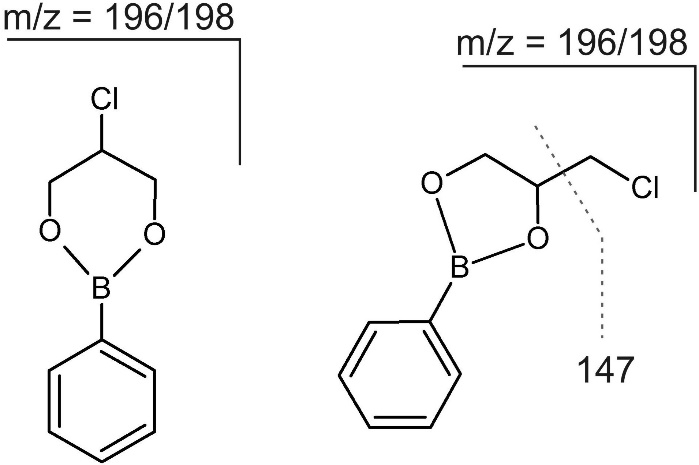


**Figure S2.** Ions of the dioxaborolane derivative of 2-MCPD (left) and 3-MCPD (right) detected by GC-MS analysis in the SIM mode. The targets of the method were the intact mother ion of the 2-MCPD derivative (*m/z* = 196) and the derivative of 3-MCPD after in-source fragmentation of CH_2_Cl leading to a signal of *m/z* = 147.

| **Table S1.** Parameters for the mass spectrometric detection of DHP-Val-FTH resulting from the FITC-mediated cleavage of modified Val residues from Hb and of the respective isotope-labeled standard compound DHP-d_7_-Val-FTH | | | | | | | | |
| --- | --- | --- | --- | --- | --- | --- | --- | --- |
| **analyte** | **RT** | **transition** | **Q1** | **Q3** | **DP** | **EP** | **CE** | **CXP** |
|  | **min** |  | **m/z** | **m/z** | **V** | **V** | **V** | **V** |
| DHP-Val-FTH | 12.03 | **quantifier** | 563.3 | **503.2** | **215** | **10** | **51** | **10** |
|  |  | qualifier1 |  | 447.0 | 215 | 10 | 56 | 10 |
|  |  | qualifier2 |  | 489.1 | 215 | 10 | 55 | 10 |
| DHP-d_7_-Val-FTH | 11.98 | **quantifier** | 570.3 | **503.2** | **215** | **10** | **51** | **10** |

| **Table S2.** Validation parameters for the analysis of DHP-Val in human erythrocyte samples.*^a^* | |
| --- | --- |
| **parameter** |  |
| **linear detection range*^b^* (nmol/L)** | 0.25 – 250 |
| **LOQ (nmol/L)** | 0.5 |
| **LOQ (pmol/g Hb)*^c^*** | 0.71 |
| **relative recovery*^d^* (%)** | 68.9 ± 7.7 |
| **intraday precision (%)*^e^*** | 9.1, 5.3, 9.0 |
| **interday precision (%)*^e^*** | 6.4, 10.2, 8.7 |
| *a* Due to the background of DHP-Val in human erythrocyte samples, the validation parameters were determined using the respective isotope-labeled compound DHP-d_7_-Val-FTH.  *b* The lower limit of the linear range marks the lower limit of detection (LOD).  *c* The lower limit of quantification (LOQ, pmol/g Hb) was calculated with the standard parameters of 35 mg Hb used for the Edman degradation and a final sample volume of 50 *µ*L.  *d* Recovery of the sample preparation; mean values and SD of six samples.  *e* The coefficients of variation (CV, %) of five (interday precision) or six (intraday precision) independent analyses at three different concentration levels, corresponding to the respective LOQ, 5 x LOQ and 25 x LOQ. | |

| **Table S3.** Validation parameters for the analyses of 2-MCPD and 3-MCPD in human urine samples. | | | |
| --- | --- | --- | --- |
| **parameter** | **2-MCPD** | **3-MCPD** |  |
| **linear detection range*^a^* (r^2^)** | 2 – 100 µg/L (0.9991) | 0.5 – 100 µg/L (0.9992) |  |
| **LOD*^a^* (µg/L)** | 0.12 | 0.1 |  |
| **LOQ*^a^* (µg/L)** | 0.3 | 0.25 |  |
| **relative recovery*^a^* (mean, %)** | 98 | 99 |  |
| **intraday precision*^b^* (%)** | 4.6 | 4.4 |  |
| **interday precision*^b^* (low conc., high conc., %)** | 15, 4.3 | 15, 5.2 |  |
| **trueness*^c^*** | 93 - 104 % | 96 - 107 % |  |
| *a* Aliquots of a blank urine samples were spiked with 2-MCPD and 3-MCPD at different concentrations. The LOD and LOQ were defined by a signal/noise ≥ 3 and a signal/noise ≥ 9, respectively. The relative recoveries of 2-MCPD and 3-MCPD (86 % to 103 %) were determined from the nominal spiking levels.  *b* The intraday precision was calculated as the coefficient of variation (CV, %) from the relative recovery. The interday precision of 2- and 3-MCPD analysis (CV) was determined by 5-fold analysis of two naturally contaminated human urine samples (low concentrations: 2-MCPD 0.26 µg/L; 3-MCPD 0.24 µg/L; high concentrations: 2-MCPD 1.69 µg/L, 3-MCPD 1.77 µg/L).  *c* Due to a lack of certified reference materials for the analysis of monochloropropanediols in human urine, three certified FAPAS® soy sauce proficiency testing materials (FAPAS® 2645, 2648, 2650) were mixed with human blank urine (1:4) and analyzed as described. | | | |
